# Microcanonical and Canonical Ensembles for fMRI Brain Networks in Alzheimer’s Disease

**DOI:** 10.3390/e23020216

**Published:** 2021-02-10

**Authors:** Jianjia Wang, Xichen Wu, Mingrui Li

**Affiliations:** 1 School of Computer Engineering and Science, Shanghai University, Shanghai 200444, China; xichenwu@shu.edu.cn; 2 Shanghai Institute for Advanced Communication and Data Science, Shanghai University, Shanghai 200444, China; 3 Department of Computer Science, University of York, York YO10 5GH, UK; ml1652@york.ac.uk

**Keywords:** brain network entropy, canonical ensemble, microcanonical ensemble

## Abstract

This paper seeks to advance the state-of-the-art in analysing fMRI data to detect onset of Alzheimer’s disease and identify stages in the disease progression. We employ methods of network neuroscience to represent correlation across fMRI data arrays, and introduce novel techniques for network construction and analysis. In network construction, we vary thresholds in establishing BOLD time series correlation between nodes, yielding variations in topological and other network characteristics. For network analysis, we employ methods developed for modelling statistical ensembles of virtual particles in thermal systems. The microcanonical ensemble and the canonical ensemble are analogous to two different fMRI network representations. In the former case, there is zero variance in the number of edges in each network, while in the latter case the set of networks have a variance in the number of edges. Ensemble methods describe the macroscopic properties of a network by considering the underlying microscopic characterisations which are in turn closely related to the degree configuration and network entropy. When applied to fMRI data in populations of Alzheimer’s patients and controls, our methods demonstrated levels of sensitivity adequate for clinical purposes in both identifying brain regions undergoing pathological changes and in revealing the dynamics of such changes.

## 1. Introduction

Network neuroscience has been proved to be a sophisticated way to study the intrinsic connectivity in the brain [1]. By mapping the network structure to the neuronal activities between different brain regions, the resulting network characterisations have been demonstrated an effective and efficient way to analyse clinical disorders of the brain, such as Alzheimer’s disease (AD) [2]. Tools derived from network science have been extensively used in the analysis of brain networks, particularly those describing the functional connectivity obtained by using neuroimaging fMRI [3,4]. For example, the analysis of network entropy on the edges of a brain network provides a novel way of identifying the salient features of brain connections, which in turn can be used to distinguish patients suspected to be in the early stages of Alzheimer’s disease from healthy controls [5].

Although there is converging evidence that the application of tools in the network science, pattern recognition and machine learning can be used to solve therapeutically intractable health problems in the brain, several methodological issues have arisen that provide obstacles to the analysis of fMRI networks in the diagnosis of and study of Alzheimer’s disease [6,7]. The first and fundamental step is to create a network connectivity matrix for different anatomical regions in the brain. The nodes in these networks are usually the cortical or subcortical grey matter regions with anatomical borders visible in fMRI. The connection between the structural or functional regions is aggregated into an adjacency matrix for the brain network [1,8].

To remove inconsistent or weak interactions, the functional connectivity in the fMRI networks is usually thresholded to give matrices with binary elements [9]. This raises the technical concern about the best practical way to find the optimal threshold. One way is to set this threshold to a constant value which results in a very sparse binary adjacency matrix for the fMRI network. The networks generated have a variable number of edges in different fMRI images [10]. Another way to the threshold is to retain a constant percentage of the strongest connections, which generates a fixed number of edges in different fMRI networks. Since there is no consistent or widely agreed best method for the brain network construction, it remains a controversial issue in the study of functional connectivity in fMRI brain studies [1,7]. There are several literatures attempts to find the specific thresholds to map the fully-connected correlation matrix in a sparse binary matrix. For example, percolation analysis provide a set of hierarchically organized modules in brain to keep the strength of weak ties [11,12]. By ranking the correlations in increasing order, the global organization of the network unveils the intrinsic stability of certain number of connected components after the removal of links [12]. The choosing of a high or a small threshold determines the density of the network, and reveals the potential size of the largest component of connected regions in the brain [11].

Tools from statistical mechanics derived from thermal physics have been extensively used to provide an appropriate way of constructing and analysing fMRI networks [13]. According to this viewpoint, by mapping the nodes or edges in a network to the particles in a thermal system, ensemble methods can be used to derive the macroscopic network properties in the network from an underlying microscopic characterisation [5,14]. For the fundamental microscopic network property is the degree distribution over the nodes, the preferential attachment mechanism proposes a intuitive attempt to connect the two disciplines of the degree in the network and the energy in thermal physics [15]. Since the nodes with high degree have the larger probability to connect other nodes, this rule can be analogous to the high energy of molecules to attract others in the molecular collisions in a gas. The physical intuition of this assumption is that the structural description of a network is straightforward performed by measuring the nodal degree. The process of connections in the nodes can be representative of statistical properties by the Boltzmann distribution with a certain physical interaction encapsulated in the energy. For networks with unit edge weights (unweighted graphs), the edge connection state for each node can be mapped to the energy of each particle in the thermal system. The corresponding energies constitute the discrete microscopic states for the network [16]. From an ensemble perspective, they describe the individual microscopic states to which statistical mechanical tools, such as the partition function, can be used to derive macroscopic characterisations of the network [17].

Similar work in our previous study reveals that by analogy with the virtual particles as the network edges, the thermodynamic quantities describe the network characterisations in weighted and unweighted networks [18]. Here we propose an alternative definition of particles in the statistical ensembles that the network nodes are analogous to particles and the energy for each node is the degree [19]. We study two kinds of statistical ensemble, namely, the microcanonical ensemble and the canonical ensemble, and use these to describe the corresponding generated fMRI networks [20,21]. In physics, the microcanonical ensemble is used to describe a group of thermal systems each with the same fixed energy [22]. For brain network construction, this corresponds to a fixed fractional threshold where each created fMRI network has an identical number of edges. The canonical ensemble, on the other hand, usually describes a set of thermal systems exchanging energy with a heat bath. This physical system can be mapped to fMRI networks with a fixed value of the threshold, and where the generated networks have a variable number of edges [23]. With the appropriate ensemble description in hand, thermodynamic properties, such as temperature, Helmholtz free energy, and entropy can be used to capture the macroscopic characteristics of the network [24,25]. Here the partition function depending on temperature and the energy states plays a powerful role in describing the behaviour of the network degree distribution [26]. The variance of degree distribution and the decomposition of entropy on each node effectively are salient features that can be used in identifying the influential regions in the brain [27]. These, in turn, can be used to distinguish different groups of patients according to the degree of progression of in Alzheimer’s disease.

## 2. Materials

### 2.1. Data Acquisition

The fMRI image of all participants were obtained from the Alzheimer’s Disease Neuroimaging Initiative (ADNI) dataset. We select 687 subjects, where 193 patients were classified as healthy control patient (HC), 240 subjects as Early Mild Cognitive Impairment (EMCI), 149 subjects as Late Mild Cognitive Impairment (LMCI), and 105 as Alzheimer’s disease (AD). The selected criteria to classify between EMCI and LMCI subjects are described in the ADNI procedure manual (http://www.adni-info.org/ accessed on February 2017). A subject can present more fMRI acquisitions taken at different time steps. In our study, for each patient we choose only one acquisition (mean). Subjects’ demographic information are summarized in Table 1.

In the ADNI study, rs-fMRI data were collected yearly at baseline, one, and two-year follow-ups (three time points in total). The rs-fMRI imaging data scans take advantage of simultaneous multi-slice acceleration for echo-planar images templates with the following parameters: slice thickness = 3.3 mm, matrix = 256 × 256, spatial resolution = 3 × 3 × 3 mm${}^{3}$, number of volumes = 140, and number of slices = 48. Each image volume is acquired every two seconds with Blood-Oxygenation-Level-Dependent (BOLD) signals.

### 2.2. Data Preprocessing

We perform image pre-processing for all rs-fMRI data using a standard pipeline, including brain skull removal, slice time correction, motion correction, spatial smoothing, and temporal pre-whitening using FSL FEAT software package (http://fsl.fmrib.ox.ac.uk/fsl/fslwiki/FEAT accessed on December 2003). Specifically, the acquired rs-fMRI images are corrected for the acquisition time difference among all slices. All images are then aligned to the first volume for motion correction and a brain mask is also created from the first volume. At last, the global drift removal and band pass filtering between 0.01 Hz–0.1 Hz are performed using tool in [28]. The pre-processing steps of the T1-weighted data include brain skull removal and tissue segmentation into gray matter (GM), white matter (WM), and cerebrospinal fluid (CSF) using FSL FAST software package (http://fsl.fmrib.ox.ac.uk/fsl/fslwiki/FAST accessed on December 2003). The pre-processed T1 image is then co-registered to the first volume of the preprocessed rs-fMRI data of the same subject and the BOLD signals in GM are merely extracted and adopted to avoid the relatively high proportion of noise caused by the cardiac and respiratory cycles in WM and ventricle [29]. Finally, the whole brain of each subject in rs-fMRI space is parcellated into 90 regions of interest (ROI), by warping the automated anatomical labeling (AAL) template [30] to the rs-fMRI image space of each subject using the FSL FLIRT software package (http://fsl.fmrib.ox.ac.uk/fsl/fslwiki/FLIRT accessed on July 2009). For each of the 90 ROIs, the mean rs-fMRI time series was calculated by averaging the GM-masked BOLD signals among all voxels within the specific ROI.

### 2.3. Brain Network Construction

We use Pearson correlation coefficients to build functional connectivity between the ROIs. Specifically, for each subject, we construct a fully connected functional connectivity network, where each node corresponds to a particular ROI and the edge weight is the Pearson correlation coefficient of a pair of specific ROIs. Then, we apply Fisher’s r-to-z transformation on the elements of the functional connectivity network to improve the normality of the correlation coefficients.

An fMRI network in the microcanonical ensemble has a fixed number of nodes and edges. The generated cross-correlation coefficients between the pairs of ROIs are thresholded to give a fixed fraction of edges. The threshold is chosen to select the largest 30% of the cumulative cross-correlation distribution, and thus to provide an optimistic edge bias for constructing fMRI networks.

An fMRI network in the canonical ensemble has a variable number of edges with a fixed number of nodes. This generates a cross-correlation network for each patient with a different number of connections between ROIs. Here the constant value of the threshold is set to be 0.8, again so as to generate optimal connections in the fMRI networks.

## 3. Methods and Procedure

In this paper, we apply ensemble methods from statistical physics to analyse fMRI brain networks for Alzheimer’s patients. By mapping the nodes in a network to virtual particles in a thermal system, the microcanonical ensemble and the canonical ensemble are analogous to two different fMRI network representations. These representations are obtained by selecting a threshold on the BOLD time series correlations between two nodes in different ways. The microcanonical ensemble corresponds to a set of networks with a fixed fraction of edges, while the canonical ensemble corresponds to the set networks with edges obtained with a fixed value of the threshold. In the former case, there is zero variance in the number of edges in each network, while in the latter case the set of networks have a variance in the number of edges. Ensemble methods describe the macroscopic properties of a network by considering the underlying microscopic characterisations which are in turn closely related to the degree configuration and network entropy. Our treatment allows us to specify new partition functions for fMRI brain networks, and to explore a phase transition in the degree distribution. The resulting method turns out to be an effective tool to identify the most salient anatomical brain regions in Alzheimer’s disease and provides a tool to distinguish groups of patients in different stages of the disease.

### 3.1. Preliminaries

Let G(V,E) be an unweighted and undirected network with a set of nodes *V* and a set of edges E⊆|V|×|V|. The adjacency matrix *A* is defined as

(1) $$\begin{array}{c} A=\left\{\begin{array}{cc} 1 & \mathrm{if}\;(u,v)\in E\\ 0 & \mathrm{otherwise}. \end{array}\right. \end{array}$$

where (u,v) is a pair of nodes forming an edge in the network. The corresponding degree matrix *D* is diagonal, where the elements are the degrees of the nodes,

(2) $$D\left(u,u\right)=d_{u}=\sum_{v\in N}A_{uv}$$

For a weighted network $G_{w}$, the pair of nodes (u,v) contains a real non-negative value w(u,v) for each edge, i.e., u∈V,v∈V, and u≠v. The adjacency matrix $A_{w}$ for a weighted network is given by

(3) $$\begin{array}{c} A_{w}=\left\{\begin{array}{cc} w(u,v) & \mathrm{if}\;(u,v)\in E\\ 0 & \mathrm{otherwise}. \end{array}\right. \end{array}$$

where, for the undirected network, the weighted adjacency is symmetric, i.e., w(u,v)=w(v,u) for all pairs of nodes that (u,v)∈E,u≠v.

### 3.2. Statistical Ensembles

Gibbs originally introduced the concept of the ensemble to describe the microscopic properties of thermal systems [31]. Here, we apply this definition to use two different statistical ensembles in the representation of brain functional connectivity networks [32].

- **The microcanonical ensemble.** This is an ensemble of networks which have a fixed number of nodes and edges. Each edge has an unit weight. This gives a preliminary definition of energy and entropy that associate with the network structure.
- **The canonical ensemble.** This is an ensemble of networks which have a fixed number of nodes but a variable number of edges. Each edge has the unit weight. This allows us to introduces the concept of temperature, associated with the variance of the number of edges. The degree of each node is analogous to the energy states of the thermal system.

#### 3.2.1. Microcanonical Ensemble

In the microcanonical ensemble, a network is regarded as an isolated system with a fixed number of both nodes |V| and edges |E|. The nodes in the network are mapped to the particles in the thermal system [33]. The corresponding node degrees are analogous to the discrete energy states. Thus, the occupation number of the energy states depends on the degree of the nodes connected by edges.

The probability distribution for individual node at the energy state can be given by the exponential function in the microcanonical ensemble

(4) $$P_{s}=\frac{1}{Z}e^{-\beta E_{s}}$$

where *Z* is the partition function following the constrain of energy conservation

(5) $$Z=\sum_{s=0}^{\left|V-1\right|}e^{-\beta E_{s}}$$

where $E_{s}$ is the possible energy state for each node in the network. For the unweighted network with unity edge weight, $E_{s}=0,1,2,...,\left|V-1\right|$. Then, the average energy can be derived from the corresponding partition function

(6) $$\bar{U}=-\frac{1}{Z}\frac{\partial Z}{\partial \beta }=-\frac{\partial \log Z}{\partial \beta }$$

The related entropy in the network can also be calculated from partition function

(7) $$S=-\sum_{s=0}^{\left|V\right|}P_{s}\log P_{s}=\beta \bar{U}+\log Z$$

This provides a framework to describe a network in the microcanonical ensemble with the thermal quantities, such as partition function, energy and entropy.

#### 3.2.2. Canonical Ensemble

Similar to the microcanonical ensemble, networks in the canonical ensemble have the fixed number of nodes but a variable number of edges. In this case, the total number of edges in a network is longer a constant. From Equation (7), the change of entropy with respected to the change of energy is

(8) $$dS=\beta d\bar{U}+\bar{U}d\beta +\frac{\partial \log Z}{\partial \beta }d\beta =\beta d\bar{U}$$

Then the definition of temperature or equivalently the parameter β i.e., the inverse temperature, is related to the rate of change of energy with respect to entropy of the network, is

(9) $$T={\left(\frac{\partial U}{\partial S}\right)}_{\left|V\right|}=\frac{1}{k_{B}\beta }$$

where $k_{B}$ is Boltzmann constant, β is the inverse temperature.

This illustrates how the various number of edges relates to the total entropy in the network structure, which also reflects the relationship between the average degree and network entropy.

Then, the Helmholtz free energy with temperature is given by

(10) $$F=\bar{U}-TS=-T\log Z$$

The corresponding entropy in Equation (7) can also be derived from the Helmholtz free energy

(11) $$S=-{\left(\frac{\partial F}{\partial T}\right)}_{\left|V\right|}={\left[\frac{\partial (T\log Z)}{\partial T}\right]}_{\left|V\right|}$$

Thus, all of the thermal quantities are related to the partition function and temperature, which describe the degree distribution and the total number of edges in the network.

### 3.3. Microscopic Quantities in Nodes

Here we commence by considering a network in the microcanonical ensemble. Each edge weight *w* is unity. By mapping the nodes in a network to the particles, the energy per node is proportional to the degree of each node, that is

(12) $$E_{u}=d_{u}\cdot w=kw$$

where w=1 for an unweighted network. k∈Z which is a positive integer or zero and equal to the number of edges connecting to the node *u*.

A network in the microcanonical ensemble has a fixed number of nodes |V| and edges |E|. Its entropy can be computed using Boltzmann’s law $=k_{B}\log W\left(U\right)$, where W(U) is the multiplicity of states and the total energy in the network is

(13) U=w|E|

which is an integer number being equal to the total number of edges when the weight is unity.

The multiplicity of states W(|V|,U) relates to the number of ways for choosing |E| edges among the available U+|V|−1 positions. Commencing from a single node in the network with *s* state, i.e., W(1,s)=1, we can derive a function to generate the series as

(14) $$\sum_{s=0}^{\infty }W\left(1,s\right)\cdot t^{s}=\sum_{s=0}^{\infty }t^{s}=\frac{1}{1-t}$$

where |t|<1 is a temporary parameter will not appear in the final multiplicity expression.

For a network with |V| nodes, the generating function is

(15) $${\left(\frac{1}{1-t}\right)}^{\left|V\right|}={\left(\sum_{s=0}^{\infty }t^{s}\right)}^{\left|V\right|}=\sum_{s=0}^{\infty }W\left(|V|,s\right)\cdot t^{s}$$

because the number of ways a term $t^{s}$ can appear in the |V|-fold product, is precisely the number of ordered ways in which the integer *s* can be formed as the sum of |V| non-negative integers. We derive that

(16) $$\begin{array}{cc} W(|V|,s) & =\lim_{t\to 0}\frac{1}{s!}{\left(\frac{d}{dt}\right)}^{s}\sum_{s=0}^{\infty }W\left(|V|,s\right)\cdot t^{s}\\ \; & =\lim_{t\to 0}\frac{1}{s!}{\left(\frac{d}{dt}\right)}^{s}{\left(1-t\right)}^{-|V|}\\ \; & =\frac{1}{s!}\left|V|(|V|+1)(|V|+2)\cdots (|V|+s-1\right) \end{array}$$

This is given by the combinatorial formula in terms of the factorials

(17) $$W\left(|V|,U\right)=\frac{(U+|V|-1)!}{U!(|V|-1)!}$$

When number of nodes and edges are large, The entropy relates the expression logW(U) can be simplified by using Stirling’s approximation logn!≈nlogn−n and as a result

(18) $$\begin{array}{cc} S & =k_{B}\ln W\\ \; & =\log[(U+|V|-1)!]-\log(U!)-\log[(|V|-1)!]\\ \; & =(U+|V|-1)\log(U+|V|-1)-U\log U-(|V|-1)\log(|V|-1) \end{array}$$

where $k_{B}$ is the Boltzmann constant.

From the definition of temperature in Equation (9), the inverse temperature β is

(19) $$\beta ={\left(\frac{\partial S}{\partial U}\right)}_{\left|V\right|}=\frac{1}{w}\log\frac{U+|V|-1}{U}$$

Then the exponential term of β is related to the average degree when |V|≫1

(20) $$e^{-\beta w}=\frac{w|E|}{w|E|+|V|-1}\approx \frac{w\bar{d}}{w\bar{d}+1}$$

The derived temperature in Equation (19) can also be extended to the networks in the canonical ensemble. The network establishes an equilibrium temperature, so that the thermodynamic partition function in Equation (5) can be represented as a serial expansion

(21) $$\begin{array}{c} Z=\sum_{k=0}^{\left|V\right|}g_{k}e^{-\beta kw}=\frac{1-e^{-|V|\beta w}}{1-e^{-\beta w}}\approx \frac{1}{1-e^{-\beta w}} \end{array}$$

where the factor $g_{k}$ is the degeneracy multiplicity of the energy state. To simplify the calculation, we assume the degeneracy factor to unity and the number of nodes in a network tends to infinity.

In Equation (4), the probability of each node at a given energy state depends on the nodal degree

(22) $$P\left(d_{u}=k\right)=\frac{1}{Z}e^{-\beta E_{s}}=\left(1-e^{-\beta w}\right)e^{-\beta kw}$$

This gives a formula for the distribution of degree in terms of thermodynamic temperature β. From Equation (20). The exponential term is controlled by temperature and depends on the total number of nodes and edges in the network. Substituting into Equation (22), the degree distribution can be rewritten as

(23) $$\begin{array}{c} P\left(d_{u}=k\right)=\frac{|V|-1}{w|E|+|V|-1}{\left(\frac{w|E|}{w|E|+|V|-1}\right)}^{k} \end{array}$$

Instead of describing the network using macroscopic thermal quantities, here we attempt to explore the microscopic characterisations for nodes. Equation (20) gives the relationship between the average degree and the inverse temperature as

(24) $$\bar{d}=\frac{\left|E\right|}{\left|V\right|}=\frac{1}{e^{\beta w}-1}$$

Then, the nodal variance in the degree can be computed as

(25) $$\begin{array}{cc} \bar{{\left(\Delta d\right)}^{2}} & =\frac{1}{\left|V\right|}\sum_{u=1}^{\left|V\right|}{\left(d_{u}-\bar{d}\right)}^{2}=\frac{1}{\left|V\right|}\sum_{u=1}^{\left|V\right|}{\left(d_{u}-\frac{1}{e^{\beta w}-1}\right)}^{2} \end{array}$$

This provides a statistical feature for each node which allows us to quantify how much the degree of node deviates from the average when the network is in the thermal equilibrium.

When the total number of nodes in the network is large, the approximate partition function in Equation (20) can be used to compute the expected variance of the degree

(26) $$\bar{{\left(\Delta d\right)}^{2}}=\frac{\partial^{2}\log Z}{\partial \beta^{2}}=\frac{w^{2}e^{\beta w}}{{\left(1-e^{\beta w}\right)}^{2}}$$

Therefore, both the nodal probability description and the degree variance can be used as microscopic features in the network. These two characterisations can be derived from the macroscopic partition function and temperature in the statistical ensembles.

### 3.4. Discriminant Analysis in Classification

Finally, we apply the discriminant analysis by considering samples of brain networks with the features of degree variance and the nodal entropy. Here, we combine both of nodal degree and entropy as the ordered components of a feature vector for that network. Since the brain networks have the fixed number of ROIs, we focus on the network collections with the same number of vertices.

Suppose there are groups of brain network with *n* samples. Each of the brain networks belongs to different *C* classes. Let $K_{c}$ be the index-set of a group of networks with combined features belonging to the class *c*, and let ${\vec{f}}_{i}$ be the feature vector of each brain network with the index *i*. The mean value of features for each class is given by

(27) $$\mu_{c}=\frac{1}{|K_{c}|}\sum_{i\in K_{c}}{\vec{f}}_{i}$$

and the average value of the overall population is

(28) $$\mu =\frac{1}{n}\sum_{i=1}^{n}{\vec{f}}_{i}$$

Thus, the between class covariance matrix for the edge brain feature vector is equivalent to

(29) $$B=\frac{1}{n}\sum_{c=1}^{C}\left(\mu_{c}-\mu \right){\left(\mu_{i}-\mu \right)}^{T}$$

The corresponding within-class variance *W*, on the other hand, is given by

(30) $$W=\frac{1}{n}\sum_{c=1}^{C}\frac{1}{|K_{c}|}{\hat{X}}_{c}{\hat{X}}_{c}^{T}$$

where $X_{c}$ is the matrix with feature vectors for class c as columns.

For jointly maximising the between-class covariance and minimising the within-class variance, we use the joint criterion

(31) $$J=\frac{u^{T}Bu}{u^{T}Wu}$$

This separation criterion is maximised by the eigenvectors *u* of the matrix $W^{-1}B$ when the separation criterion will be equal to the corresponding eigenvalue.

If $W^{-1}B$ is diagonalizable, the variability between feature vectors will be contained in the subspace spanned by the eigenvectors corresponding to the C−1 largest eigenvalues. These eigenvectors can be used in feature reduction, as in principal component analysis. The eigenvectors corresponding to the smaller eigenvalues will tend to be very sensitive to the exact choice of training data, and it is often necessary to use regularisation.

For network classification, we apply support vector machine (SVM) to classify different groups of patients with brain network features. These features extracted from the eigenvectors in discriminant analysis associated with the eigenvalues falling into the top 10 percentile. The discriminant analysis model is based on the assumption that the edge features follow a multivariate normal distribution with an identical covariance matrix for each class.

By applying SVM in classification, the algorithm attempts to find the best hyperplane with the largest margin between the two classes. The separating hyperplane identifies the closest feature points, known as support vectors, to find the boundary of classification. When consider the binary separation between AD and NC groups, the problem is equivalent to find the optimal solution in hyperplane that enables classification of a vector *z* as follows

(32) class(x)=sign(k·x+b)=sign(f(x))

where *x* is the set of feature points, $k\in R^{n}$ is the parameter in hyperplane, *b* is a real number, f(x) is the classification score and represents the distance *x* is from the decision boundary. This can be solved by using Lagrange multipliers to find the optimal value in *k* and *b* to find the best hyperplane in classification.

## 4. Experimental Results

In this section, we apply the proposed ensemble methods to investigate the fMRI networks. We first explore whether the nodal entropy can identify specific inter-regional connections and regions in the brain associated with the neurodegeneration caused by the onset of Alzheimer’s disease. Then, we apply the derived microscopic and macroscopic characterisations to analyse fMRI network structure and distinguish different groups of Alzheimer’s patients.

### 4.1. Salient ROI Detection

**Figure 1:**
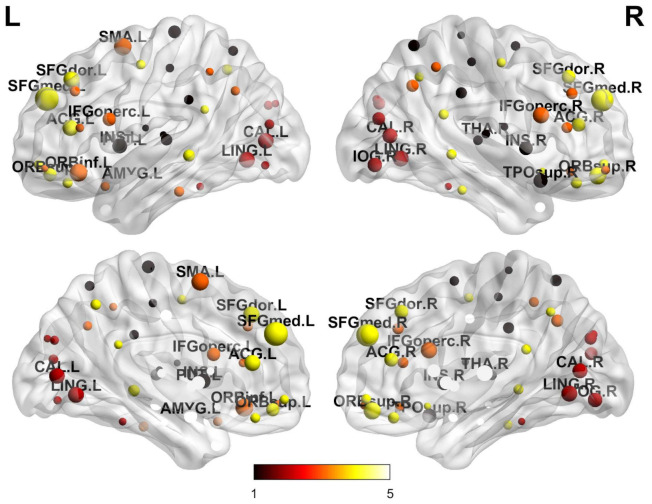
The significant different of nodal entropy in the anatomical regions in the brain.

To determine which anatomical regions play the most significant role in the development of Alzheimer’s disease, we use the derived nodal entropy to identify the differences in the brain regions. Here, we compute the standardized Euclidean distance, and apply the *p*-value in the *T*-test for each nodal entropy between two populations, i.e., AD and NC. The large value of Euclidean distance in entropy, with the *p*-value less than 0.01, identify the significant difference in brain regions between normal health group and Alzheimer’s disease. Figure 1 plots the most significant nodal entropies for the anatomical regions of the brain. Patients with the depressive neurodegenerative disease have structural and functional inhibition in the frontal lobe and occipital lobe [34,35]. They are severely damaged by Alzheimer’s disease with aberrant symptoms that affect recognition, memory and emotional behaviour [34].

Table 2 lists the top ten anatomical regions by the difference in nodal entropy. These include the Superior Frontal Gyrus, Inferior Frontal Gyrus, Supplementary Motor Area, Lingual Gyrus, Thalamus, etc. This is in line with related clinical research. For example, the behavioural symptoms in AD-associated with specific frontal cortical areas [36].

### 4.2. Alzheimer’s Classification

**Figure 2:**
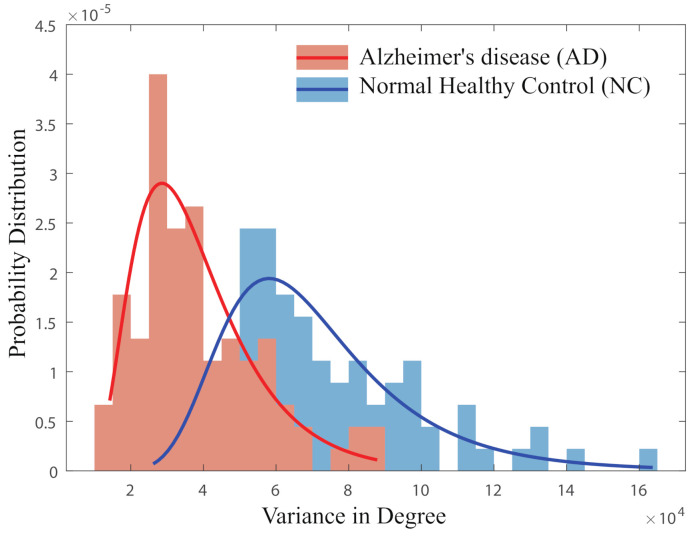
Probability distribution of degree variance in AD and NC groups.

Taking this analysis further, we explore whether the degree variance in the microcanonical ensemble and the nodal entropy in the canonical ensemble can be combined as features to classify patients in the Alzheimer’s disease study. We first consider the node degree variance in the microcanonical ensemble networks. Since brain networks in this category have the same number of edges and nodes, the values of temperature are identical for all fMRI networks. To distinguish the fMRI network structures in AD and normal people, we plot the distribution of degree variance in Figure 2. This shows that the brain networks in AD occupy the lower range of node degree variance compared to the normal subjects.

**Figure 3:**
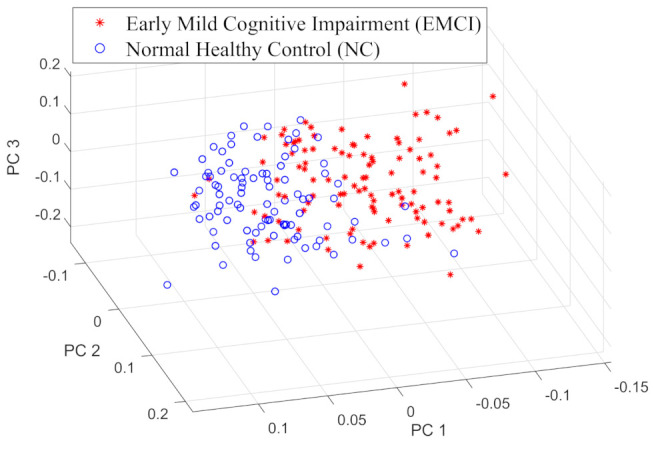
3D visualisation of the principal components of node entropy between groups of Normal Healthy Control (NC) and Early Mild Cognitive Impairment (EMCI).

An important clinical and neurological issue is the determination of the early symptoms in Alzheimer’s disease. To this end, we apply the combined feature vector from the two canonical ensembles to distinguish the structural difference in fMRI networks between patients in the AD and EMCI categories. The fMRI network for each subject has an associated feature vector with degree variance and node entropy as components capturing the structural feature of the network. Figure 3 is the 3D visualisation of the projection of the feature vectors onto the non-orthogonal eigenvectors of Fisher’s discriminant in the linear discriminant analysis(LDA). We project the fMRI network feature vectors onto the three principal (leading) eigenvectors, and these show distinct clusters for each group of patients. The salient feature is that the entropy features for the nodes are effectively working as network characterisations to separate EMCI subjects from the other groups of AD patients.

Finally, we take the projected feature vectors (analogous to principal components) as the characterisation for each fMRI brains network and then apply SVM (Support Vector Machine) with Gaussian kernel to classify data into four groups of patients. The 687 patients in the dataset are randomly separated into training data (500 samples) and testing data (154 samples). The training and testing accuracies are shown in Table 3 after 10 fold cross-validation (the random assignment to test and training data is randomised 10 times and the results averaged). For the binary classification, as an example, we randomly divide the AD and NC subjects into 10 disjoint subsets of equal size. Remove one subset, train the classification model using the other nine subsets. This process is repeated by removing each of the ten subsets once at a time and then average the classification accuracy.

For the four categories of patients, the total classification accuracy is 82.35%. For the binary classification between Early Mild cognitive impairment (EMCI) and healthy control (NC), the accuracy drops slightly to 71.43% but is still excellent, and allows us to distinguish early patients form the normal health group. The binary classification accuracy between full Alzheimer’s disease (AD) and the normal healthy control (NC) groups is 80.61%. Thus, the resulting method combined with the network characterisations from fMRI connectivity networks works as an efficient tool to identify patients suspected as suffering from Alzheimer’s disease.

In conclusion, both the degree variance in the microcanonical ensemble and the nodal entropy in the canonical ensemble are useful to characterise the fMRI brain networks. The synthetic analysis suggests that there exists a phase transition with the value of temperature in both structural characterisations. The analysis of real-world datasets demonstrates these derived structural features are powerful to distinguish different fMRI brain networks in Alzheimer’s disease.

## 5. Discussion

**Figure 4:**
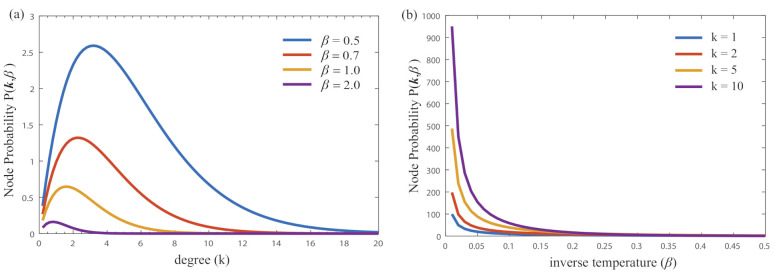
The node probability varying with the degree *k* and inverse temperature β in Equation (21). (**a**) node probability with degree; (**b**) node probability with inverse temperature.

We first conduct a numerical analysis on the node probability in Equation (22). Figure 4 plots how the node probability varies with the degree *k* and inverse temperature β, respectively. In Figure 4a, there is a phase transition for the probability varying with the node degree. When the value of inverse temperature β increases, the peak corresponding to the phase transition shifts towards zero. In Figure 4b, the node probability exponentially decays with the inverse temperature. The larger value of node degree, the faster in decay.

**Figure 5:**
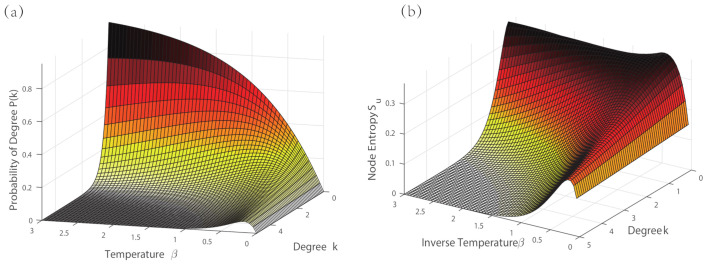
(**a**) 3D plot of degree probability with different value of degrees *k* and the inverse temperature β; (**b**) 3D plot of node entropy with different value of degrees *k* and the inverse temperature β.

Then, we investigate the degree probability distribution given in Equation (22), which relates to the inverse temperature β and the degree of a node $d_{u}=k$. Figure 5a shows a three-dimensional plot of dependence between the three quantities. For a small value of the nodal degree, the degree probability decreases monotonically by reducing the inverse temperature β. While for high degree nodes, the degree probability presents a slight peak in the high-temperature region, but still remaining at a low value of probability. This maximum illustrates that a transition has occurred in the degree distribution with the inverse temperature, and depends on the value of degree at the nodes.

Similarly, we analyse the relationship among the entropy of the nodes, the inverse temperature and the degree. We again plot a three-dimensional visualisation in Figure 5b. Each node entropy in the network decreases as the degree (or the number of edge connections) increases. This means the larger degree, the lower the value of entropy at each node. In terms of the temperature, there is a peak that is similar to that observed in the degree probability in the high-temperature region. Thus, there is also a phase transition for the entropy at each node with a varying value of temperature.

Finally, we make a comparison to the state-of-the-art methods in Alzheimer’s classification. Here, we use the directed degree and von neumann entropy in our previous methods as the brain network features to classify different groups of patients [37]. Table 4 shows the corresponding results. For the directed degree features, although the testing accuracies in binary classification of AD/NC and EMCI/NC are slightly better the current method, the overall accuracy for four groups cannot reach at the performance of statistical ensemble method. This is because the directed degree features in the network are more affected by the threshold value of network construction; while the thermal quantities from statistical ensembles propose a more general way of constructing fMRI network which is less affected by the threshold parameter.

When we apply the von Neumann entropy to distinguish different brain networks, Table 4 shows that the average classification accuracy for both training and testing cases is around 75%. This is about 15% lower than when our proposed thermal characterisations are used. Therefore, the corresponding methods to characterise fMRI networks can be used to identify patients with early onset of Alzheimer’s disease in the clinical application.

The advantages of this our proposed methods are twofold. One is the construction of brain networks. This provides a better understanding of the statistical connections in the brain among different groups of patients. Networks built from microcanonical and canonical ensembles propose a new way to understand how the brain’s structural wiring supports the mental health treatments. Another is the merit of feature selection which will improve the performance of classifier. The proposed measures related to specific nodes in the brain identify the most influenced regions in Alzheimer’s pathology. This provides the most informative features to make the best classification by reducing a high volume of data to a small salient set. The clinical meaning is to provide a powerful tool to detect the early Alzheimer’s disease from the healthy subjects.

## 6. Conclusions

In this paper, we present a novel way to analyse fMRI networks from the statistical ensembles. Two kinds of ensemble networks, i.e., microcanonical ensemble and canonical ensemble, are studied and suggest different ways of choosing the activation thresholds in fMRI network generation. Networks in the microcanonical ensemble have the same number of edges, while the networks in the canonical ensemble have variable numbers of edges. The corresponding ensemble methods describe the macroscopic characterisations of the network from the microscopic properties. The microscopic energy states in the thermal system are analogous to the degree of nodes with the unit edge weight. This derives the definition of temperature and partition function used to characterise the structural properties in the network. The degree distribution presents a phase transition with the value of temperature. By applying the resulting methods, we analyse the fMRI networks in Alzheimer’s disease. Each kind of ensemble method relates to a way of choosing certain kinds of threshold in the binary functional activation network constriction. With an expression for the degree distribution to hand, we decompose the global network entropy into contributions associated with each node and use this to identify the most affected anatomical regions in the brain. The variance of associated node degree combined with node entropy work well as the features to classify different groups of patients.

Although preliminary results suggest the effectiveness of our methods, we recognize that our theoretical analysis and experimental results are not definitive. Future work will focus on the description of a grand-canonical ensemble for a network and will explore different ways of segmenting regions in the brain. The second line of investigation will investigate the distribution of weights on the edges, which describe the distribution of energy states, instead of the current assumption based on the discrete distribution with unit edge weights. A further line of investigation would be to explore the possibility of a strong interaction between pairs of nodes without restricting the nodes in the networks to be distinguishable and weakly interacting.

## Figures and Tables

**Table 1:** Demographics and neuropsychological data for all groups of patients (in Gender, M is Male and F is Female, and SD is Standard Deviation).

Group	Number of Patients	Gender	Age Range (Years)	Mean Age (SD)
HC	193	80M/113F	65–96	73.42 (±7.2)
EMCI	240	100M/140F	56–91	73.67 (±7.2)
LMCI	149	109M/58F	57–90	73.69 (±7.2)
AD	105	47M/58F	56–89	73.48 (±7.3)

**Table 2:** Top 10 significant different ROIs in AD and NC groups.

AAL Index	ROI Name	R/L	Abbreviation
23	Superior frontal gyrus, (Medial)	Left	SFGmed
24	Superior frontal gyrus, (Medial)	Right	SFGmed
15	Inferior frontal gyrus, (Orbital part)	Left	ORBinf
19	Supplementary motor area	Left	SMA
6	Superior frontal gyrus, (Orbital part)	Right	ORBsup
3	Superior frontal gyrus, (Dorsolateral)	Left	SFGdor
12	Inferior frontal gyrus, (Opercular part)	Right	IFGoperc
48	Lingual gyrus	Right	LING
47	Lingual gyrus	Left	LING
78	Thalamus	Right	THA

**Table 3:** Classification accuracy with thermal nodal quantities in SVM with 10-fold cross validation.

	Training Accuracy	Testing Accuracy
AD/NC	85.50% (171/200)	80.61% (79/98)
EMCI/NC	83.00% (249/300)	71.43% (95/133)
Total Four Categories	90.60% (453/500)	82.35% (154/187)

**Table 4:** Classification accuracy in SVM with 10-fold cross validation.

		Training Accuracy	Testing Accuracy
Directed Degree	AD/NC	82.00% (168/200)	84.69% (83/98)
	EMCI/NC	82.00% (246/300)	84.21% (112/133)
	Total Four Categories	86.10% (431/500)	73.80% (138/187)
Von Neumann Entropy	AD / NC	68.50% (137/200)	67.35% (66/98)
	EMCI/NC	70.67% (212/300)	61.65%(82/133)
	Total Four Categories	75.60% (378/500)	71.12% (133/187)
Thermal Quantities	AD/NC	85.50% (171/200)	80.61% (79/98)
	EMCI/NC	83.00% (249/300)	71.43% (95/133)
	Total Four Categories	90.60% (453/500)	82.35% (154/187)

## Data Availability

Publicly available datasets were analyzed in this study. This data can be found here: http://www.adni-info.org/.
