# Erratum: Wang, J., et al. Microcanonical and Canonical Ensembles for fMRI Brain Networks in Alzheimer’s Disease. *Entropy* 2021, *23*, 216

**DOI:** 10.3390/e23040438

**Published:** 2021-04-09

**Authors:** Jianjia Wang, Xichen Wu, Mingrui Li, Hui Wu, Edwin R. Hancock

**Affiliations:** 1 School of Computer Engineering and Science, Shanghai University, Shanghai 200444, China; xichenwu@shu.edu.cn; 2 Shanghai Institute for Advanced Communication and Data Science, Shanghai University, Shanghai 200444, China; wuhui616@shu.edu.cn; 3 Department of Computer Science, University of York, Heslington YO10 5GH, UK; ml1652@york.ac.uk (M.L.); edwin.hancock@york.ac.uk (E.R.H.)

We sincerely apologize for the inconvenience of updating the authorship. The previous authorship was Jianjia Wang, Xichen Wu, Mingrui Li, and the updated authorship is Jianjia Wang, Xichen Wu, Mingrui Li, Hui Wu, Edwin R. Hancock. Ms. Hui Wu and Professor Edwin R. Hancock should be added to the list of contributing authors. Ms. Hui Wu contributed to the formal analysis, methodology, and investigation in the original manuscript. Professor Edwin R. Hancock contributed to the supervision, methodology, and the review and editing of the manuscript. We have added them to the authorship of our paper [1] and updated their contributions in acknowledgement. The updated “Author Contributions” is provided below:
